# Molecular structure and the role of high‐temperature requirement protein 1 in skeletal disorders and cancers

**DOI:** 10.1111/cpr.12746

**Published:** 2019-12-22

**Authors:** Yihe Li, Jinbo Yuan, Emel Rothzerg, Xinghuo Wu, Huazi Xu, Sipin Zhu, Jiake Xu

**Affiliations:** ^1^ Department of Orthopaedics The Second Affiliated Hospital and Yuying Children's Hospital of Wenzhou Medical University Wenzhou China; ^2^ Division of Regenerative Biology School of Biomedical Sciences University of Western Australia Perth WA Australia; ^3^ Department of Orthopedics Union Hospital Tongji Medical College Huazhong University of Science and Technology Wuhan China

**Keywords:** cancer, cartilage, gene, high‐temperature requirement protein 1, molecular

## Abstract

Human high‐temperature requirement protein 1 (HTRA1) is a member of serine proteases and consists of four well‐defined domains—an IGFBP domain, a Kazal domain, a protease domain and a PDZ domain. HTRA1 is a secretory protein and also present intracellularly and associated with microtubules. HTRA1 regulates a broad range of physiological processes via its proteolytic activity. This review examines the role of HTRA1 in bone biology, osteoarthritis, intervertebral disc (IVD) degeneration and tumorigenesis. HTRA1 mediates diverse pathological processes via a variety of signalling pathways, such as TGF‐β and NF‐κB. The expression of HTRA1 is increased in arthritis and IVD degeneration, suggesting that HTRA1 protein is attributed to cartilage degeneration and disease progression. Emerging evidence also suggests that HTRA1 has a role in tumorigenesis. Further understanding the mechanisms by which HTRA1 displays as an extrinsic and intrinsic regulator in a cell type–specific manner will be important for the development of HTRA1 as a therapeutic target.

## 1 INTRODUCTION

Human high‐temperature requirement protein 1 (HTRA1), also known as L56, or serine protease 11 was first isolated from human fibroblasts as a putative serine protease.^1^ HTRA1 belongs to the HTRA superfamily with characteristics of a serine protease and plays a role in ATP‐independent protein quality control. Current findings indicate that HTRA1 is not only present extracellularly as a secretory protein, but also found intracellularly and is associated with microtubules to regulate cell migration.^2^ HTRA1 takes part in many biological processes and cellular signalling pathways, and is implicated in pathogenesis including osteoporosis, osteoarthritis, Alzheimer's disease, age‐related macular degeneration and cancer.^3^, ^4^, ^5^, ^6^ This topical review discusses recent findings pointing to the role of HTRA1 in bone biology, arthritis and tumorigenesis. In addition, this review also provides an update of the molecular structure of HTRA1 and its underlining mechanisms of action in these disorders. Advancing the understanding of the role of HTRA1 and other HTRA family members may help us develop HTRA1 as a therapeutic target.

## 2 GENE ANALYSIS, MOLECULAR STRUCTURE AND FUNCTION OF HTRA1

**Figure 1:**
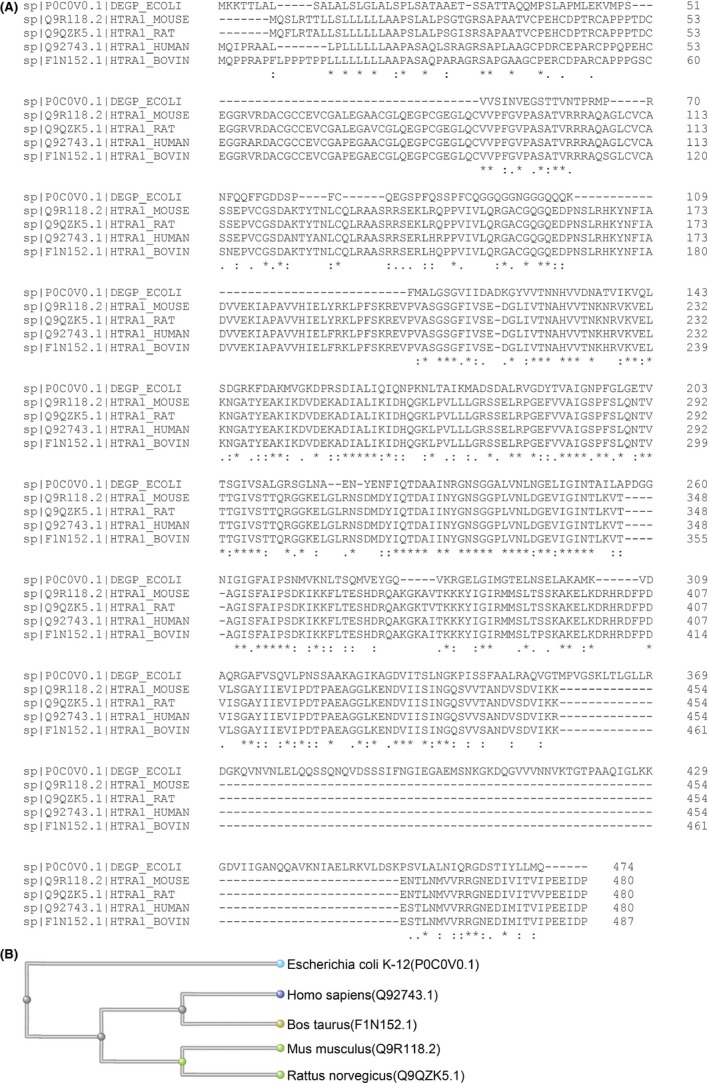
HTRA1 amino acid sequence analysis. A, Multiple amino acid sequence alignment analysis showing the similarities and differences in HTRA1 homologs among human, mouse, rat, bovine and *Escherichia coli*. B, Phylogenetic tree analysis indicating that human HTRA1 is most closely related to bovine HTRA1

**Figure 2:**
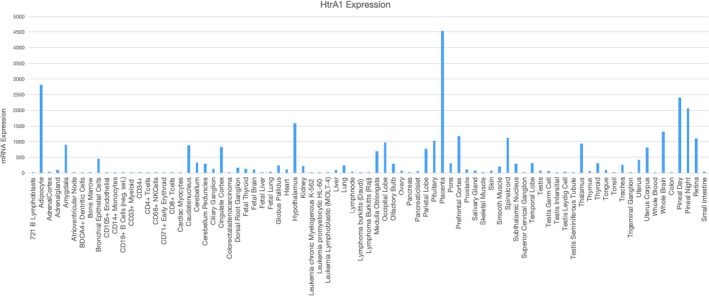
BioGPS analysis of HTRA1 expression in different human tissues and cells showing that HTRA1 is most abundantly expressed in the placenta and adipocyte. Analyses were performed based on the raw data in BioGPS (http://ds.biogps.org/)

Human HTRA1 gene is located at 10q26.13. Multiple alignment analysis shows similarities and differences in HTRA1 homologues among human, mouse, rat, bovine and *Escherichia coli* (Figure 1A). Human HTRA1 is closely related to bovine HTRA1 according to phylogenetic tree analysis (Figure 1B). Gene expression analysis by BioGPS indicates that *HTRA1* is ubiquitously expressed among various human tissues and cells, and most highly expressed in the placenta and adipocyte (Figure 2).

**Figure 3:**
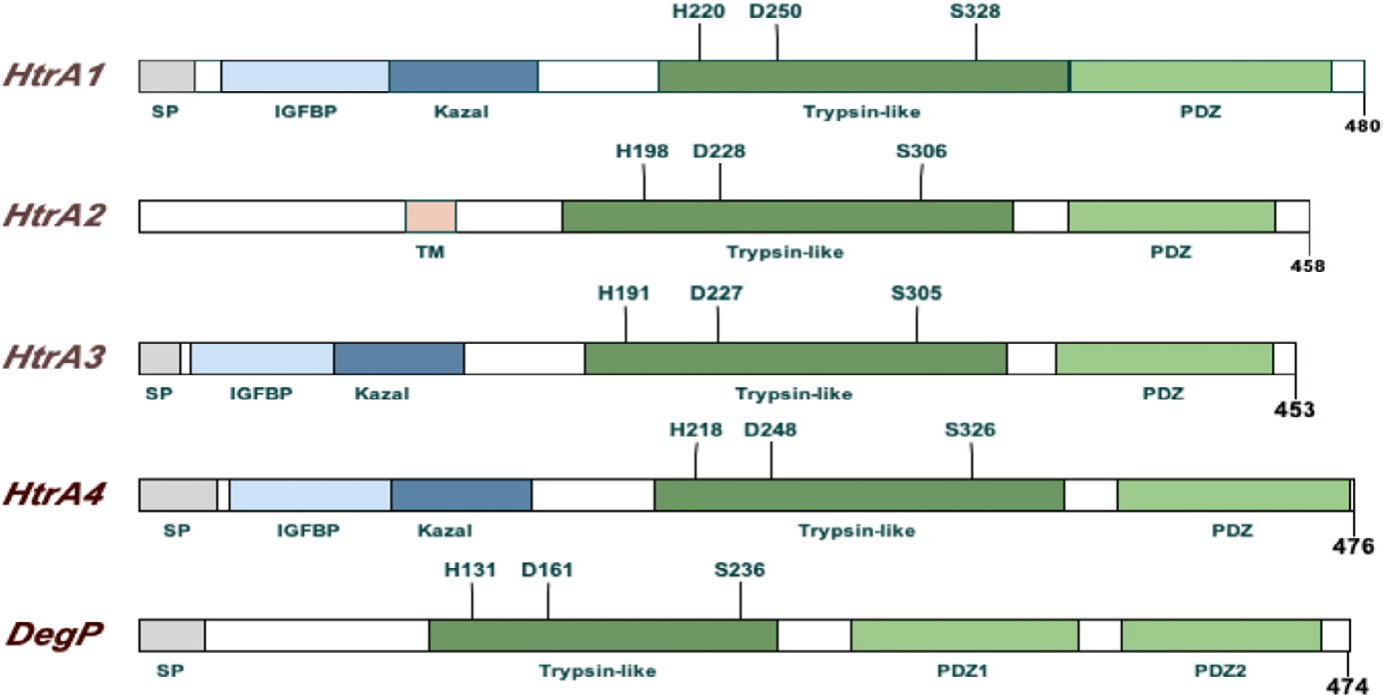
Domain structure of human HTRA 1‐4 and bacterial HtrA/DegP. Human HTRA and bacterial HtrA share same trypsin‐like serine protease domain and PDZ domains. Human HTRA 1, 3 and 4 also share the same N‐terminus, and HTRA2 contains a transmembrane domain (TM)

HTRA1 is a trypsin‐like serine protease. Bacterial and mammalian HtrA genes share common structural features with a highly conserved trypsin‐like serine protease domain and one or two PDZ domains at the carboxyl terminus. Different from other HtrA family proteins, human HTRA1, 3 and 4 consist of an amine terminal (N‐terminal) signal peptide, an IGFBP domain, PDZ domain and a Kazal domain (Figure 3).^7^

HTRA1 was identified as a secretory protein and also found present intracellularly.^2^, ^8^ HTRA1 belongs to a member of chymotrypsin‐like protease, which can be switched between active and inactive conformation.^9^, ^10^ The activation mechanism of human HTRA1 is still unclear, and a suggested mechanism refers that HTRA1 is self‐regulated via inter‐monomer communication.^11^ HTRA1 plays a role in protein quality control, by which it recognizes aberrant proteins and cleaves them into smaller fragments, thereby preventing protein misfolding and mislocalization.^10^ Through its proteolytic activity, HTRA1 is involved in a variety of physiological processes and disease pathogenesis. The trypsin‐like serine protease domain in human HTRA1 is responsible for proteolytic activity, whereas the PDZ domain and the N‐terminal domain bear no relation to its proteolytic activity.^9^, ^10^, ^11^, ^12^ The N‐terminal domain of human HTRA1 contains an IGFBP domain and a Kazal domain, which may mediate the autolysis of HTRA1 protein via acting as a redox‐sensing switch.^9^ In bacterial HtrA, the PDZ domain is required for allosteric regulation, recognition of the unfolded protein, activation of the enzyme and degradation of the substrate.^13^ In human HTRA1, PDZ domain is required for the processing of polymeric proteins, such as tubulin and amyloid fibril, and is associated with microtubules to mediate cell migration.^2^, ^6^, ^11^, ^14^

## 3 THE POTENTIAL ROLE OF HTRA1 IN BONE FORMATION AND RESORPTION

**Figure 4:**
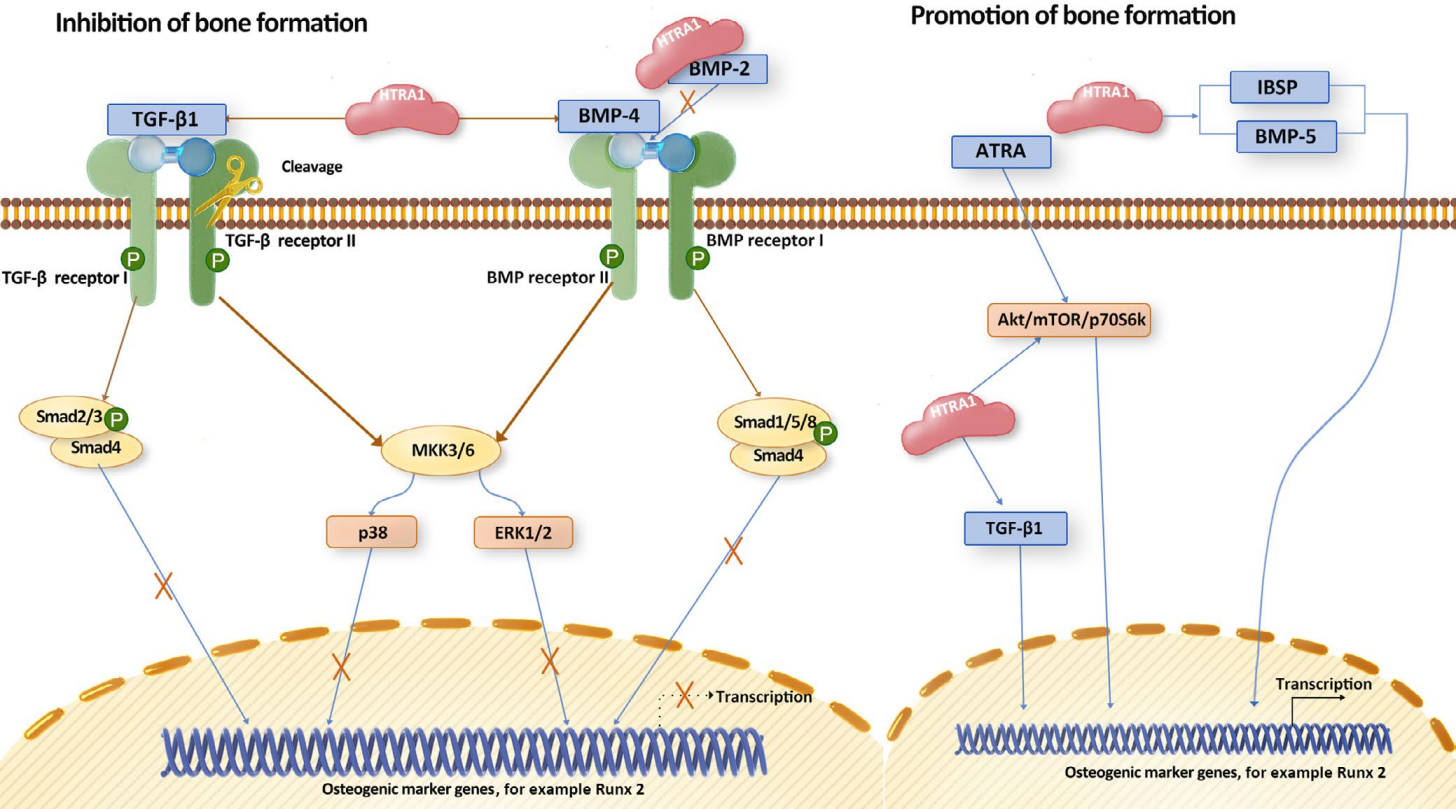
Proposed direct role of HTRA1 in bone formation. HTRA1 regulates TGF‐β/BMP signalling pathway, and ATRA signalling in osteoblastic cells. HTRA1 appears to play a dual effect in inhibition or promotion of bone formation

Bone development and growth continue until early adulthood. The dynamic balance between bone formation by osteoblasts and bone resorption by osteoclasts is vital in maintaining bone microstructure and density.^15^, ^16^ The imbalance between osteogenesis and osteoclastogenesis leads to bone diseases such as osteoporosis and osteopetrosis.^15^, ^16^ Bone formation is known to be regulated by several major signalling pathways, including BMP and TGF‐β. TGF‐β and BMP induce Smad‐dependent signalling pathway and Smad‐independent signalling cascade, such as MKK3/6.^17^ Accumulating evidence points to a role of HTRA1 in bone formation via its regulation in TGF‐β and BMP signalling molecules (Figure 4).

HTRA1 is secreted by both osteoblasts and osteoclasts,^3^, ^18^, ^19^ and act as an inhibitory molecule of bone formation through suppressing the activity of the TGF‐β/BMP pathway.^3^, ^20^ The inhibitory effect of HTRA1 on TGF‐β/BMP is dependent on its proteolytic activity via degrading TGF‐β family proteins, such as BMP‐2, BMP‐4 and TGF‐β1, and cleaving TGF‐β receptors.^20^, ^21^ Furthermore, we have found that HTRA1 inhibits bone formation via attenuating BMP‐2–induced phosphorylation of Smad1/5/8, ERK1/2 and p38.^19^ Consistently, HTRA1 knockout mice have been shown to display increased trabecular bone mass and higher bone density.^21^, ^22^

On the other hand, few studies have reported that HTRA1 plays a positive role in bone formation.^23^, ^24^, ^25^ HTRA1 promotes formation of mineralized matrix and enhances osteogenesis in human bone marrow‐derived mesenchymal stem cells (hBMSCs), human periodontal ligament cells (hPDLCs) and mouse adipose‐derived stromal cells (mASCs).^23^, ^24^, ^25^ In mASCs, all‐trans retinoic acid (ATRA) is an inducer of osteogenesis via activation of Akt/mTOR/p70S6K pathway (Figure 4). Loss of HTRA1 has shown the inhibitory effect of ATRA via inactivation of p70S6K.^23^ In hBMSCs, HTRA1 has been found to be up‐regulated during osteogenic differentiation and to increase the mineralized matrix formation.^25^ Moreover, HTRA1 induces the expression of BMP‐5 and IBSP, which positively regulate matrix mineralization.^25^ In hPDLCs, overexpression of HTRA1 promotes osteogenesis via inducing the expression of TGF‐β1.^24^ In addition, a study in CARASIL (also known as cerebral autosomal recessive arteriopathy with subcortical infarcts and leukoencephalopathy) has found that the absence of HTRA1 in vivo is associated with decreased TGF‐β signalling.^26^ These conflicting results implicate that further investigation is required for a better understanding of the role of HTRA1 in the regulation of bone formation and TGF‐β/BMP signalling in a cell type–dependent manner.

**Figure 5:**
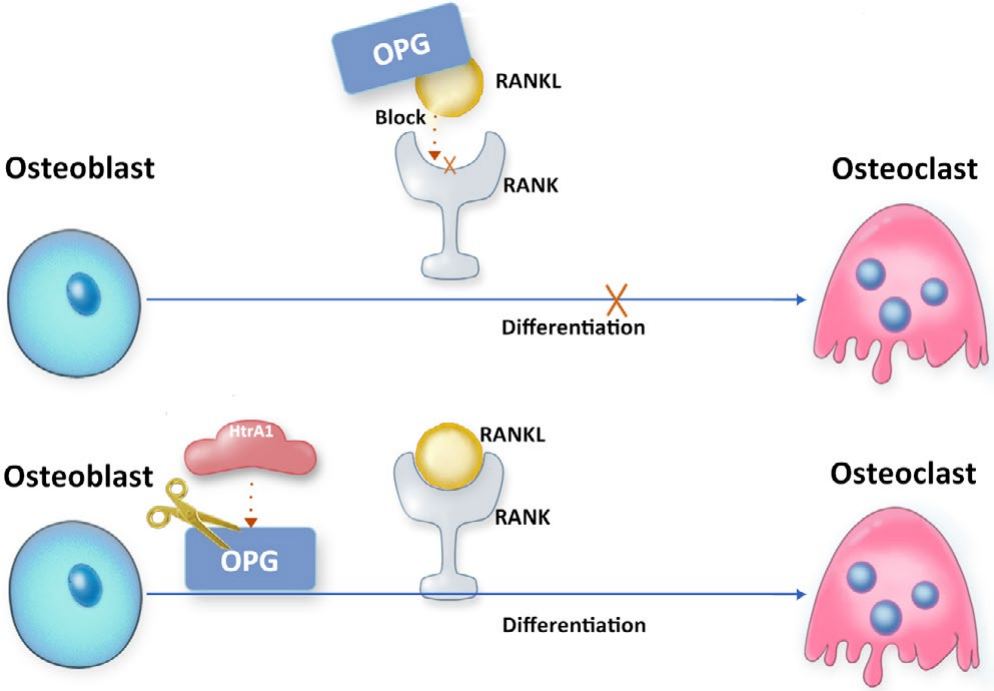
Proposed indirect role of HTRA1 in bone resorption via RANKL/OPG axis. HTRA1 activates RANKL‐induced osteoclastogenesis involved in the degradation of OPG, a decoy receptor of RANKL

In bone resorption, HTRA1 plays a role through the regulation of osteoprotegerin (OPG) (Figure 5). OPG, as a decoy receptor of RANKL is a suppressor of osteoclastogenesis via inhibiting RANKL‐induced osteoclast differentiation. A recent study conducted by Ochiai et al^18^ has indicated that HTRA1 is secreted by osteoclasts, acts as OPG‐degrading enzyme and inhibits OPG activity during RANKL‐induced osteoclastogenesis (Figure 5). Further, osteoclast‐secreted HTRA1 has been found to be up‐regulated during RANKL‐induced osteoclastogenesis,^19^ which might also attenuate OPG activity in bone microenvironment.

Collectively, the role of HTRA1 in the regulation of bone formation and bone resorption is related to TGF‐β/BMP signalling, which appears to be dose and stage‐dependent.^27^, ^28^ Some inconsistent results regarding the effects of HTRA1 on bone formation may be due to complex interactions between HTRA1 and TGF‐β family proteins in different cells and dosages of HTRA1 used in each experimental design. Further understanding the role of HTRA1 in bone formation and bone resorption might help to develop HTRA1 a therapeutic target in bone metabolic diseases such as osteoporosis.^22^

## 4 THE ROLE OF HTRA1 IN ARTHRITIS AND DISC PATHOLOGY

Osteoarthritis (OA) is a degenerative joint disease, and primary risk factors for OA include ageing, obesity and joint injury.^29^, ^30^ Rheumatoid arthritis (RA) is an autoimmune disease, caused by the immune system that falsely attacks the healthy synovium tissue in joints. Although the mechanisms of OA and RA are different, they share a common pathological feature—the destruction of cartilage in the joint.^29^, ^31^

**Figure 6:**
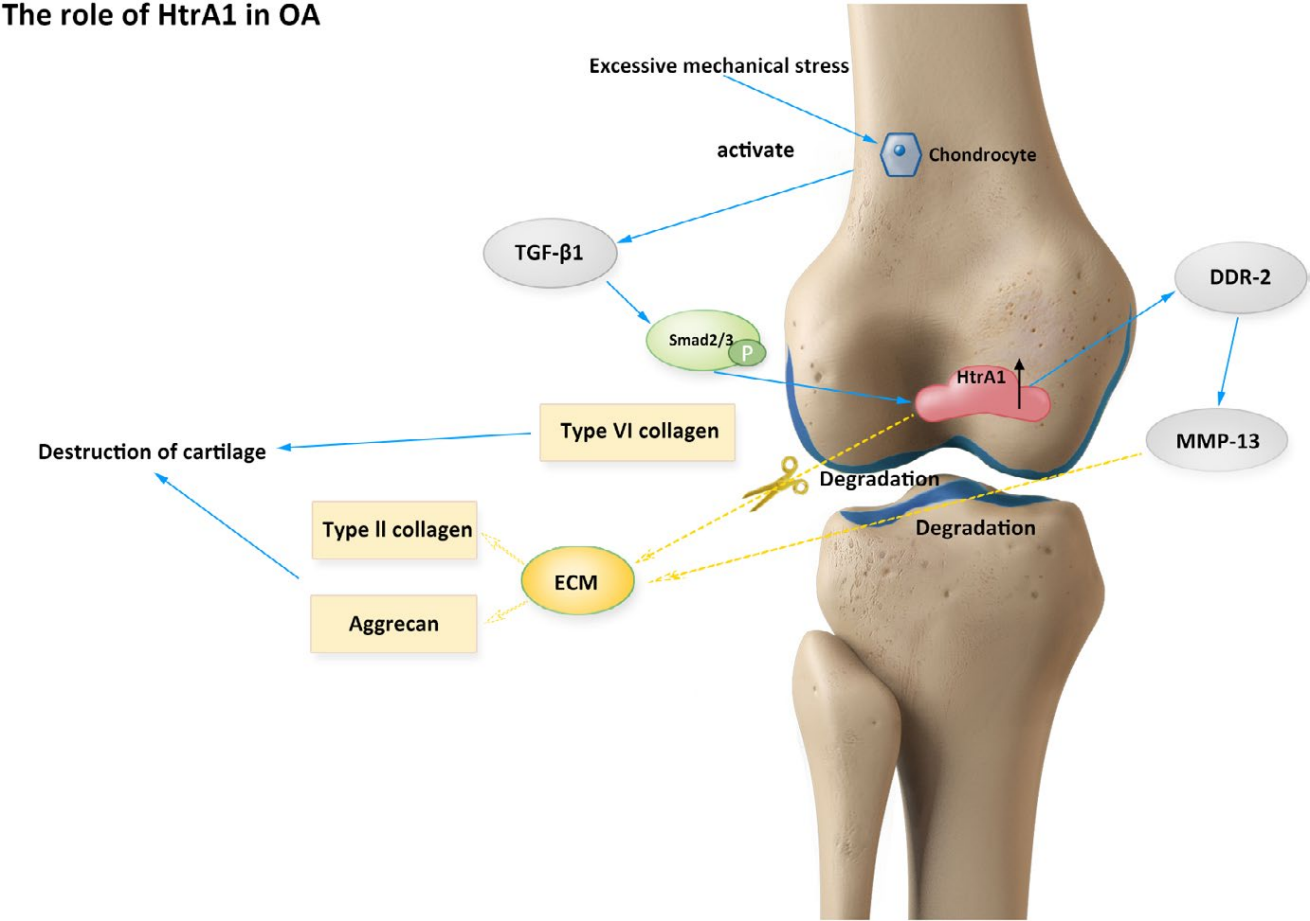
Proposed role of HTRA1 in OA. HTRA1 is up‐regulated in cartilage tissues and associated with DDR2, MMP‐13 and TGF‐β1 in the regulation of OA progression

HTRA1 is an emerging mediator of arthritic diseases and has been found up‐regulated in synovial fluid and articular cartilage tissues in patients with OA and RA.^4^, ^32^ HTRA1 is involved in the interaction with discoidin domain receptor 2 (DDR‐2), MMP‐13 and TGF‐β proteins (Figure 6).^33^, ^34^, ^35^, ^36^ Elevated HTRA1 expression was found during OA development and related with DDR‐2 activation.^36^ DDR‐2 is a cell surface receptor, in which down‐regulation of DDR‐2 leads to delayed articular cartilage degradation.^37^ MMP‐13 induced by DDR2 can degrade extracellular matrix such as type II collagen and aggrecan, thus affecting OA development.^38^ TGF‐β also plays a key role in OA development.^35^, ^36^ Inactivated TGF‐β1 signal can alleviate OA progress, while increasing TGF‐β1 promotes proteolytic cleavage of ECM and downregulates type VI collagen expression in chondrocytes by inducing HTRA1 expression through phosphorylating SMAD2/3, exacerbating the progression of OA.^32^, ^33^, ^35^, ^36^ Further, HTRA1 is up‐regulated in fibroblasts and macrophages in the development of RA.^39^ In RA, IFN‐γ inhibits HTRA1 expression in joint tissue via activation of p38 MAPK/STAT1 pathway, which directly regulates the promoter of HTRA1, resulting in deceased HTRA1 transcription.^39^ More recently, it was revealed that cartilage degeneration process is significantly delayed in Htra1^−/−^ mice compared with wild‐type littermates,^40^ further indicating that HTRA1 is involved in the degradation of ECM via its proteolytic activity.

Intervertebral disc (IVD) degeneration is the most common reason for low back pain, of which risk factors include ageing, mechanical stress and other environmental factors.^41^ ECM components are comprised 70% of the dry weight of the intervertebral disc, and breakdown of ECM results in intervertebral disc degeneration. Similar to arthritis, HTRA1 has been found up‐regulated in degenerated IVD tissues and to promote IVD degeneration via breakdown of ECM.^42^ HTRA1 is involved in the degradation of ECM through inducing expression of MMPs and aggrecanases (ADAMTS) in IVD via MEK signalling pathway.^42^ Injection of HTRA1 into healthy IVD results in loss of disc height.^43^ Moreover, HTRA1 has been suggested as a potential treatment target for IVD degeneration owing to its role in the cleavage of chondroadherin (CHAD).^44^ CHAD fragment is a biomarker in disc degeneration as it was only found in degenerated IVD tissues and increased level of fragmented CHAD has been correlated with the severity of disc degeneration.^44^

## 5 THE ROLE OF HTRA1 IN CANCERS

HTRA1 is emerging as a cancer stromal and a tumour suppressor gene.^45^ In 2001, Shidhar et al^46^ found that HTRA1 is down‐regulated in the malignant ovarian tumour cells. Subsequent studies have also reported that HTRA1 expression is decreased in different types of cancers, including metastatic cancers.^47^, ^48^, ^49^, ^50^, ^51^, ^52^ An early study on ovarian cancer has suggested a high frequency of loss of heterozygosity near *HTRA1* may be associated with its decreased expression in cancer cells.^49^ Many signalling pathways in tumour progression are regulated by HTRA1 via proteolysis. For example, HTRA1 cleaves fibroblast growth factor‐8 (FGF‐8) protein in the extracellular region and inhibits FGF signalling pathway in neuroblastoma.^53^ Down‐regulation of HTRA1 was found to promote anchorage‐independent growth in ovarian cancer cells.^49^ HTRA1 also controls cell proliferation via inhibiting Wnt/β‐catenin signalling pathway.^54^ HTRA1 forms a complex with β‐catenin thereby inactivates the Wnt/ β‐catenin signalling and reduces the proliferation rate of cancer cells.^54^ Inhibitory effects of HTRA1 on Wnt signalling is protease‐independent and may be associated with TGF‐β signalling.^54^ Down‐regulation of HTRA1 in endometrial cancer has shown increased invasive and metastatic ability in tumour cells.^55^ HTRA1 also inhibits tumour metastasis via promoting anoikis in cancer cells.^56^ In ovarian cancer cell lines, overexpression of HTRA1 inactivates EGFR/AKT signalling pathway and induces anoikis.^56^ Moreover, HTRA1 is a microtubule‐associated protein, which mediates tubulins degradation and cell migration.^2^ HTRA1 suppresses tumour angiogenesis via activating Notch signalling pathway in tumour stroma and inducing the expression of vascular endothelial growth factor receptor‐2 (VEGFR‐2).^57^ HTRA1 is also an essential regulator of epithelial to mesenchymal transition (EMT), which is associated with tumour invasion, metastasis and drug resistance in chemotherapy.^58^, ^59^ EMT is also induced by TGF‐β signalling, and thereby, HTRA1 regulates EMT of cancer cells via inhibiting TGF‐β signalling.^20^, ^58^, ^60^ However, the mechanism by which HTRA1 expression was down‐regulated in tumour tissues remains to be elucidated.

**Figure 7:**
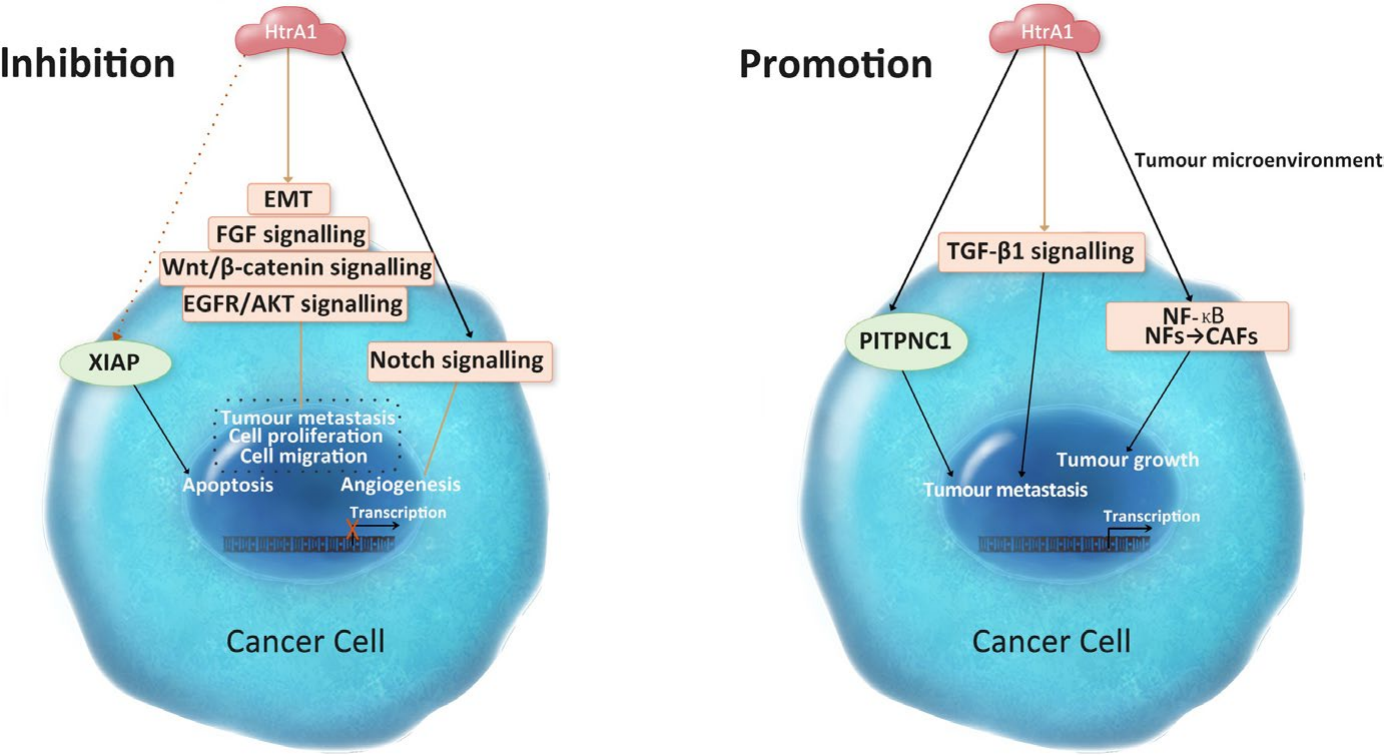
Proposed role of HTRA1 in cancer cells. HTRA1 regulates tumour progression via a variety of signalling pathways and plays a role in the tumour microenvironment

Although HTRA1 is being classified as a tumour suppressor, several studies have suggested that *HTRA1* could be a pro‐oncogenic gene.^61^, ^62^, ^63^ A previous study has suggested that HTRA1 inhibits EGFR/ART signalling pathway and induces cell death.^56^ However, a recent study highlighted that HTRA1 is bound to a tumour promoter lysyl oxidase (LOX), which can inhibit TGF‐β1 signalling, increase MATN2 expression and then enhance the expression of EGFR, thereby promoting tumour progression and metastasis.^61^ HTRA1 also acts on tumour microenvironment and promotes tumour growth via inducing cancer‐associated fibroblasts (CAFs).^62^ CAFs in tumour microenvironment positively regulate tumorigenesis, and α‐SMA is a major biomarker in CAFs.^64^ A recent study has reported that higher expression of HTRA1 in gastric cancer tissue is correlated with lower survival outcome in patients.^62^ Overexpression of HTRA1 in gastric cancer cells activates NF‐kappa B (NF‐κB) signalling pathway, thereby induces the expression of basic fibroblast growth factor (bFGF) and up‐regulation of α‐SMA.^62^ Increased expression of α‐SAM induces transdifferentiation of normal fibroblasts to CAFs.^62^ HTRA1 has also been found to be associated with the pro‐metastatic protein *PITPNC1* and promote tumour metastasis.^63^ Moreover, a cohort study in ovarian cancer has indicated that HTRA1 down‐regulation in the nucleus in cancer cells is correlated with a higher survival rate in patients with high‐grade ovarian tumour.^65^ Another study has found that in a HPV‐positive cell line, overexpression of HTRA1 promotes cell proliferation.^66^ Thus, HTRA1 has multi‐functions in tumorigenesis (Figure 7).

Cisplatin and paclitaxel are the two anti‐tumour agents that are widely used in the treatment of solid tumour. Most patients initially have a favourable response to these chemotherapeutic agents. However, drug resistance usually develops after long‐term treatment. HTRA1 increases cell sensitivity to the cytotoxicity of paclitaxel and cisplatin via degrading X‐linked inhibitor of apoptosis protein (XIAP).^67^, ^68^, ^69^ The expression level of XIAP is negatively correlated with HTRA1 expression in hepatocellular carcinoma cells.^67^ In addition, down‐regulation of HTRA1 expression in the ovarian cancer cell is related to the poor outcome of patients with chemotherapy.^68^

As mentioned before, TGF‐β is associated with HTRA1 in tumorigenesis via Wnt/β‐catenin signalling, EMT and EGFR.^56^, ^58^, ^60^, ^61^ Consistently, the TGF‐β signalling pathway can either be a tumour suppressor in the early stage of cancer cells via inducing cell death or be a tumour promoter in advanced stage of cancer development via inducing metastasis and acting on the tumour microenvironment.^60^, ^70^ In tumorigenesis, the expression of HTRA1 has been found to be negatively correlated with the expression of TGF‐β1 in endometrial cancer tissues.^51^ However, in ovarian cancer and thyroid carcinoma, no significant difference between HTRA1 and TGF‐β1 expression levels has been found.^50^, ^52^ It is likely that HTRA1 inhibits TGF‐β signalling in tumour cells at the early stage of cancer via its proteolytic activity. At the late stage, the HTRA1 level is low in cancer cells and unable to bind with TGF‐β and fails to inhibit TGF‐β signalling. Further study is required to unlock the complex interplay between HTRA1 and TGF‐β in a cell type–dependent manner, which is necessary for the understanding of HTRA1 as a multi‐functional regulator in cancers.

## 6 CONCLUSION

Human HTRA1 is a serine protease, which is widely expressed in human tissues and most abundantly expressed in the placenta and adipocyte. HTRA1 is a critical mediator in many cellular processes and plays a role in the pathogenesis of arthritis and IVD degeneration. HTRA1 also affects bone formation and bone resorption via the regulation of TGF‐β/BMP and RANKL/OPG signalling. In cancers, HTRA1 regulates a broad range of signalling in tumour microenvironment, and through which mediates cancer cell proliferation, migration and invasion. To fully understand the mechanisms of HTRA1 in diseases, further investigation of the molecular function of HTRA1 as an extrinsic and intrinsic regulator in a cell type–dependent manner is essential.

## CONFLICT OF INTEREST

No conflict of interest.

## AUTHOR CONTRIBUTION

Yihe Li conducted data research and drafted the manuscript. Jinbo Yuan provided assistance on the structural analysis of HTRA1. Emel Rothzerg, Xinghuo Wu and Huazi Xu provided valuable opinions, evaluation and assistance in the process of drafting and revision of the manuscript. Sipin Zhu and Jiake Xu co‐supervised the study, provided conceptual framework and revised the manuscript.

## Data Availability

The data that support the findings of this study are available from the corresponding author upon reasonable request.
